# Effects of Twenty-Four Weeks of Resistance Exercise Training on Body Composition, Bone Mineral Density, Functional Fitness and Isokinetic Muscle Strength in Obese Older Women: A Randomized Controlled Trial

**DOI:** 10.3390/ijerph192114554

**Published:** 2022-11-06

**Authors:** Sung-Woo Kim, Hun-Young Park, Won-Sang Jung, Kiwon Lim

**Affiliations:** 1 Physical Activity and Performance Institute, Konkuk University, 120 Neungdong-ro, Gwangjin-gu, Seoul 05029, Korea; 2 Department of Sports Medicine and Science, Graduate School, Konkuk University, 120 Neungdong-ro, Gwangjin-gu, Seoul 05029, Korea; 3 Department of Physical Education, Konkuk University, 120 Neungdong-ro, Gwangjin-gu, Seoul 05029, Korea

**Keywords:** senior fitness test, hand grip strength, dominant leg, fat-free mass, resistance band exercise

## Abstract

Resistance exercise effectively improves bone mineral density (BMD) and muscle quality (e.g., muscle mass and muscle strength). The present study aimed to examine the effect of a 24-week resistance exercise training (RT) program on body composition, BMD, functional fitness, and isokinetic muscle strength in obese older women. Forty obese older women were initially enrolled. Among them, 30 participants (age: 80.55 ± 4.94 years; body fat percentage: 36.25 ± 3.44%) completed the study. The participants were randomly assigned into two groups: the RT group (n = 15) and the control (CON) group (n = 15). The RT group participated in the exercise for 60 min per session and two sessions per week for 24 weeks. Pre-test and post-test body composition, BMD, functional fitness, and isokinetic muscle strength were evaluated. The RT group increased significantly in functional fitness (hand grip strength: 1.70 kg, *p* < 0.01, and lower body strength: 3.87 n, *p* < 0.001), and isokinetic muscle strength (non-dominant leg extensor peak torque %BW at 60°/s: 13.20%, *p* < 0.05, dominant leg (DL) flexor peak torque at 60°/s: 3.87 Nm, *p* < 0.05, and DL flexor peak torque %BW at 60°/s: 7.60%, *p* < 0.05). However, the CON group showed negative changes in body composition (fat mass: 1.15 kg, *p* < 0.001, body fat percentage: 1.59%, *p* < 0.001, and fat-free mass: −0.58 kg, *p* < 0.05), BMD (whole-body: −0.01 g/cm^2^, *p* < 0.001 and forearm: −0.01 g/cm^2^, *p* < 0.05), functional fitness (lower body flexibility: −3.23 cm, *p* < 0.01, upper body strength: −2.06 n, *p* < 0.01, and agility and dynamic balance: 0.54 s, *p* < 0.01), and isokinetic muscle strength at 60°/s and 180°/s (all peak torque % body weight variables: −7.31–−1.50, *p* < 0.05). Our findings show that the CON group negatively affects body composition, BMD, functional fitness, and isokinetic muscle strength in obese older women for 24 weeks.

## 1. Introduction

The worldwide population of individuals aged above 65 years has been increasing rapidly [1]. Aging-induced biological changes in humans negatively affect daily life due to a decrease in the function of tissues or organs [2]. In particular, physiological aging increases the risk of increased fat mass, changes in body composition due to redistribution of fat, changes in metabolism, decreased muscle mass and muscle function, cardiovascular diseases, and osteoporosis [3,4,5]. A previous study reported that older women exhibited a 10% reduction in fat-free mass and a 23% increase in fat mass due to aging when compared with younger adults [6]. These changes in body composition appear rapidly after menopause [7].

Obesity is a rapidly increasing health problem in modern society that increases the risk of chronic diseases that lead to debilitation and death [8,9]. Obese people have negative health effects due to the menopause and aging, especially worsening cardiovascular conditions, sarcopenia, accumulation of visceral adipose tissue, and reduced bone mineral density (BMD) [10]. In addition, muscle mass decreases by approximately 1–2% annually after the age of 50 years [11]. A decrease in muscle mass reduces muscle strength, thereby reducing walking ability and balance in older adults [12]. Delaying this decrease in muscle mass and muscle strength, which is associated with increasing age in older adults, plays an important role in improving the activities of daily living [13]. Osteoporosis is the most important metabolic bone disease in an aging society. It results in the weakening of bone microstructures, which lowers BMD and increases the risk of fractures [14]. Previous studies have reported annual reduction rates of 1.08% and 0.81% in femoral BMD and lumbar BMD, respectively, among adult Korean women after the age of 50 years [15]. Especially in women, BMD decreases rapidly after the menopause. Therefore, various exercises such as weight-bearing, resistance, and combined training have been proposed to prevent and treat aging-induced osteoporosis [16].

In modern society, the number of older adults with restricted physical functions increases with increased life expectancy, and health-related physical/functional fitness measurements are used to prevent diseases and to promote health [17]. Functional fitness is defined as the physical ability of older adults to work independently and safely without excessive fatigue while performing daily activities such as simple housework, walking, and hiking [18]. Functional fitness includes lower body flexibility, upper body flexibility, lower body strength, upper body strength, agility, and dynamic balance, and aerobic endurance [18]. Due to the aging society, the number of older adults with restricted physical functions is rapidly increasing, and the level of functional fitness is gradually decreasing with the consequent increase in the risk of falls, fractures, and physical disabilities [19]. Therefore, the American College of Sports Medicine recommends various exercise programs for older adults to reduce the risk factors for disease and to improve functional fitness [20].

It is well known that exercise improves physical fitness and health-related factors in older adults [20]. Resistance exercise effectively improves the metabolic rate, BMD, and muscle quality [21]. Moreover, resistant exercise is widely used as an effective prescription for older adults as well as for the general population. The most significant benefit of resistance exercise is its positive effect on falls and functional disorders, which are risk factors related to osteoporosis and sarcopenia [21]. A recent meta-analysis study reported that resistance exercise using elastic bands was effective for obese older women [22]. The present study aimed to examine the effects of 24-week resistance exercise training on body composition, BMD, functional fitness, and isokinetic muscle strength in obese older women.

## 2. Materials and Methods

### 2.1. Participants

**Figure 1:**
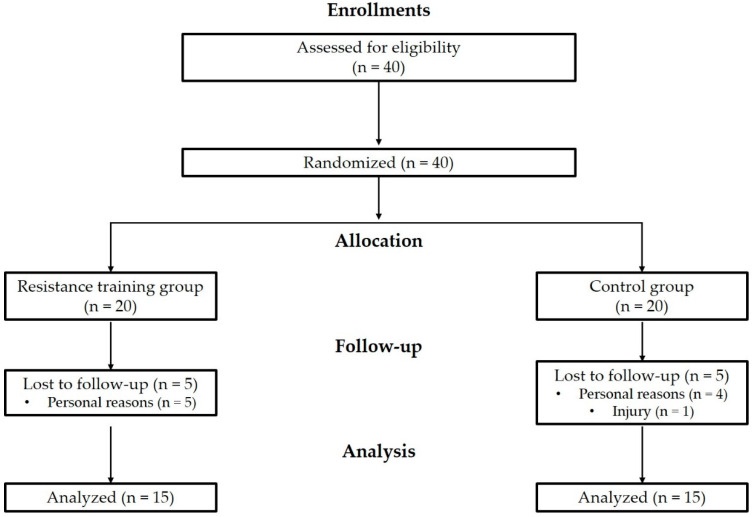
Flow chart of the study.

This experiment was conducted for 24 weeks from 19 July 2021 to 23 January 2022. Forty obese women aged 73–89 years were enrolled in this study. The inclusion criteria were participants with a body fat percentage > 30% and participants with low levels of physical activity according to the International Physical Activity Questionnaire—short form (<600 MET min/week/no exercise over the last 6 months) [23,24]. The exclusion criteria were any uncontrolled chronic diseases, a history of acute myocardial infarction, a history of joint replacement or fracture of the lower limb within the previous 6 months, and severe cognitive impairment. The participants were randomly assigned into two groups: the resistance exercise training (RT) group and the control (CON) group. However, ten participants withdrew due to personal reasons or injury. Thus, 30 participants completed the study (RT: n = 15, CON: n = 15) (Figure 1). The participant’s physical characteristics are summarized in Table 1. Using G*Power 3.1.9.2 (Franz Faul, University of Kiel, Kiel, Germany) at the power of 0.80 and effect size of 0.3, 24 was the optimal sample size when the significance level was set to 0.05. All study procedures were approved by the Institutional Review Board of Konkuk University and were conducted in accordance with the principles of the Declaration of Helsinki. The study was registered with the Clinical Research Information Service (http://cris.nih.go.kr, accessed on 25 April 2022), conforming to the World Health Organization International Clinical Trials Registry Platform (registration number: KCT0007221).

### 2.2. Resistance Exercise Training Program

The training group followed the RT program for 60 min per session (10:30 a.m. to 11:30 a.m.) and 2 sessions per week for 24 weeks [21]. The training program consisted of 10 min of warm-up (dynamic and static stretching), 40 min of RT, and 10 min of cool-down (static stretching). RT comprised abdominal curl-up, biceps curl, chest press, front shoulder raise, lateral shoulder raise, seated row, triceps extension, calf raise, chair squat, hip extension, hip flexion, standing abduction, standing adduction, and toe raise. The exercise intensity including the number of repetitions increased progressively every 4 weeks. The training intensity was set at three sets of 10–15 repetitions (yellow band: 1–4 weeks 10 rep, 5–8 weeks 12 rep, 9–12 weeks 15 rep; red band: 13–16 weeks 10 rep, 17–20 weeks 12 rep, 21–24 weeks 15 rep) at a perceived exertion value of 7 or 8 on the OMNI-Resistance Exercise Scale of Perceived Exertion (0: extremely easy to 10: extremely hard) [25,26]. This range has been reported to correspond to exercise intensity levels of 70–80% of the one-repetition maximum (1RM) with a rest period of 90 s per set [27,28,29]. The RT program was supervised and directed by a licensed bodybuilding instructor. Details of the RT program are shown in Table 2.

### 2.3. Body Composition and Bone Mineral Density

Body composition (height, weight, fat mass, fat-free mass, and body fat percentage) was measured after fasting for more than 4 h and removal of metallic materials using bioelectrical impedance analysis equipment (Inbody 770, Inbody, Seoul, Korea) from 9:00 a.m. BMD was measured using dual-energy X-ray absorptiometry with a bone densitometer (PRIMUS, OsteoSys, Seoul, Korea) [30]. All the participants were scanned at four different sites (whole-body, femur, lumbar spine, and forearm) for BMD measurements. The whole-body BMD was measured with the subject lying on the center of the examination table and with both feet rotated slightly inward, and then the shoulders and waist were stationary. The femur BMD was measured after confirming the location of the left femoral. The lumbar BMD was measured from lumbar vertebra 1 to 4. The subject was placed on the table and flexed the hips and knees by 90° to the assistive device and placed on both legs. The measurement was performed by placing the laser at a position 2, 3 cm below the navel. The forearm BMD was measured from the non-dominant forearm. A single technician performed all the measurements.

### 2.4. Functional Fitness

Functional fitness was assessed using hand grip strength, lower body flexibility, upper body flexibility, lower body strength, upper body strength, agility and dynamic balance, and aerobic endurance [18]. Muscle strength was measured twice using the grip strength of the dominant hand on a hand grip dynamometer (T.K.K.5001; Takei Co., Tokyo, Japan). The participants were instructed to stand with their right hand 45° away from the body and grip the dynamometer as strongly as possible. The highest value was recorded to the nearest 0.1 kg. Lower body flexibility was evaluated using the chair sit and reaches test. From a sitting position on the edge of a chair with one leg extended and hands reaching toward the toes, the distance (cm) (+ or −) between the extended fingers and the tip of the toe was measured. The score was recorded to the nearest 0.1 cm. Upper body flexibility was measured using the back-scratch test. The participants were in a standing position with one hand reaching over the shoulder and the other hand reaching upward in the opposite direction toward the middle of the back. The distance (cm) between the extended middle fingers (+ or −) of the two hands was measured. The score was recorded to the nearest 0.1 cm. Lower body strength was assessed using the 30 s chair stand test. The participants were instructed to sit upright in a chair with their hands crossed and placed on their chest. The number of times they could stand and sit within 30 s after the start signal was measured. Upper body strength was assessed using the 30 s arm curl test. With their feet on the floor, participants lifted dumbbells without pressing their backs or waists to the back of the chair. They performed the arm curl test by holding a 5-pound (2.27 kg) dumbbell and curling it as many times as possible within 30 s. The number of arm curls within 30 s was recorded. Agility and dynamic balance were assessed using the 8-foot up-and-go test. The participants sat on a chair, leaning back against the wall. They were instructed to get up from the chair, walk toward a cone placed 8 feet (2.44 m) away, turn around the cone, return to the chair, and sit down again as quickly as possible without running. The time required to complete this activity was measured. Aerobic endurance was evaluated using the 2 min step test. The participants were instructed to step in place repeatedly for 2 min by raising each knee midway between the patella and the iliac crest. The score was assigned based on the number of times the right knee reached the required level.

### 2.5. Isokinetic Muscle Strength

The muscle strength of the knee extensors and flexors was measured using a Biodex System 3™ dynamometer (Biodex Medical Systems, Shirley, NY, USA). Maximal voluntary concentric isokinetic torque was assessed in Nm at angular velocities of 60°/s and 180°/s. Three repetitions at 60°/s and five repetitions at 180°/s each of maximal isokinetic quadriceps and hamstring concentric contractions in the dominant leg (DL) and non-dominant leg (NDL) were performed at two different angular velocities with a 1-min interval between the trials and maximal peak torque production was recorded [31].

### 2.6. Statistical Analysis

Statistical analyses were performed using IBM SPSS Statistics, version 26.0 (IBM Corp., Armonk, NY, USA). The mean values, standard deviations and 95% confidence intervals were calculated. The normality of distribution of all dependent variables was verified using the Kolmogorov–Smirnov test. Two-way repeated-measures analysis of variance was applied to determine the group-by-time interaction effects during the intervention. If any significant interaction or main effects were observed, independent *t*-tests and paired *t*-tests were applied to analyze the statistical significance of within-group and between-group differences. The effect size was computed as partial eta-squared values (η_p_^2^; small: ≥0.01, medium: ≥0.06, large: ≥0.14) [32]. The statistical significance was set at *p* < 0.05.

## 3. Results

### 3.1. Body Composition and Bone Mineral Density

Significant group-by-time interaction effects were observed for fat mass (F = 17.205, *p* < 0.001, η_p_^2^ = 0.372), fat-free mass (F = 5.700, *p* < 0.05, η_p_^2^ = 0.164), body fat percentage (F = 28.266, *p* < 0.001, η_p_^2^ = 0.494), whole-body BMD (F = 12.385, *p* < 0.001, η_p_^2^ = 0.299), and forearm BMD (F = 6.228, *p* < 0.05, η_p_^2^ = 0.177) (Table 3). All of the variables with statistical interaction effects had a large effect size. The post-test results showed that variables with significant interaction effect had significantly changed in the CON group (fat mass: 1.15 kg, *p* < 0.001; fat-free mass: −0.58 kg, *p* < 0.05; body fat percentage: 1.59%, *p* < 0.001; whole-body BMD: −0.01 g/cm^2^, *p* < 0.001; and forearm BMD: −0.01 g/cm^2^, *p* < 0.05), while no significant change was observed in the RT group. Moreover, significant post-test differences were observed in body fat percentage (RT: 34.83 ± 4.03%, CON: 38.46 ± 2.42%, *p* < 0.05) between the RT group and the CON group.

### 3.2. Functional Fitness

Significant group-by-time interaction effects were observed for the hand grip strength (F = 15.433, *p* < 0.001, η_p_^2^ = 0.347), lower body flexibility (F = 11.479, *p* < 0.01, η_p_^2^ = 0.284), lower body strength (F = 20.154, *p* < 0.001, η_p_^2^ = 0.410), upper body strength (F = 11.202, *p* < 0.01, η_p_^2^ = 0.279), and agility and dynamic balance (F = 7.532, *p* < 0.01, η_p_^2^ = 0.206) (Table 4). All of the variables with statistical interaction effects had a large effect size. The post-test results showed that the hand grip strength (1.70 kg, *p* < 0.01) and lower body strength (3.87 n, *p* < 0.001) had increased significantly following the 24 weeks of the RT program. In contrast, lower body flexibility (−3.23 cm, *p* < 0.01), upper body strength (−2.06 n, *p* < 0.001), and agility and dynamic balance (0.54 s, *p* < 0.01) showed significant negative changes in the CON group. Additionally, significant post-test differences were observed in hand grip strength (RT: 22.90 ± 3.34 kg, CON: 19.03 ± 3.45 kg, *p* < 0.01), lower body flexibility (RT: 24.44 ± 7.98 cm, CON: 17.68 ± 6.88 cm, *p* < 0.05), and lower body strength (RT: 18.93 ± 5.69 n, CON: 15.13 ± 3.56 n, *p* < 0.05) between the RT group and the CON group.

### 3.3. Isokinetic Muscle Strength

Significant group-by-time interaction effects were observed for DL extensor peak torque (F = 6.562, *p* < 0.05, η_p_^2^ = 0.185), DL extensor peak torque % body-weight (BW) (F = 5.962, *p* < 0.05, η_p_^2^ = 0.171), NDL extensor peak torque %BW (F = 4.706, *p* < 0.05, η_p_^2^ = 0.140), DL flexor peak torque (F = 7.251, *p* < 0.05, η_p_^2^ = 0.200), DL flexor peak torque %BW (F = 8.361, *p* < 0.01, η_p_^2^ = 0.224), NDL flexor peak torque (F = 5.598, *p* < 0.05, η_p_^2^ = 0.162), and NDL flexor peak torque %BW (F = 7.429, *p* < 0.05, η_p_^2^ = 0.204) at 60°/s (Table 5). All of the variables with statistical interaction effects had a large effect size. The post-test results showed that NDL extensor peak torque %BW (13.20%, *p* < 0.05), DL flexor peak torque (3.87 Nm, *p* < 0.05), and DL flexor peak torque %BW (7.60%, *p* < 0.05) at 60°/s had increased significantly following the 24 weeks of the RT program. In contrast, DL extensor peak torque (−4.81 Nm, *p* < 0.05) and DL extensor peak torque %BW (7.31%, *p* < 0.05) at 60°/s had decreased significantly in the CON group. Additionally, significant post-test differences were observed in the DL extensor peak torque %BW (RT: 139.73 ± 48.53%, CON: 109.50 ± 31.92%, *p* < 0.05), DL flexor peak torque %BW (RT: 62.40 ± 18.48%, CON: 46.94 ± 22.51%, *p* < 0.05), NDL flexor peak torque (RT: 35.13 ± 13.60 Nm, CON: 26.19 ± 12.10 Nm, *p* < 0.05), and NDL flexor peak torque %BW (RT: 62.93 ± 26.01%, CON: 44.50 ± 23.71%, *p* < 0.05) at 60°/s between the RT group and the CON group.

Significant group-by-time interaction effects were observed for DL extensor peak torque (F = 5.902, *p* < 0.05, η_p_^2^ = 0.169), DL extensor peak torque %BW (F = 7.193, *p* < 0.05, η_p_^2^ = 0.199), NDL extensor peak torque %BW (F = 5.618, *p* < 0.05, η_p_^2^ = 0.162), DL flexor peak torque %BW (F = 4.197, *p* < 0.05, η_p_^2^ = 0.126), NDL flexor peak torque (F = 8.183, *p* < 0.01, η_p_^2^ = 0.220), and NDL flexor peak torque %BW (F = 13.009, *p* < 0.001, η_p_^2^ = 0.310) at 180°/s (Table 6). All of the variables with statistical interaction effects had a large effect size. The post-test results showed that variables with significant interaction effect at 180°/s had decreased significantly in the CON group (DL extensor peak torque: −2.19 Nm, *p* < 0.05; DL extensor peak torque %BW: −5.06%, *p* < 0.05; NDL extensor peak torque %BW: −5.06%, *p* < 0.05; DL flexor peak torque %BW: −5.81%, *p* < 0.01; NDL flexor peak torque: −2.19 Nm, *p* < 0.05; NDL flexor peak torque %BW: −5.81%, *p* < 0.01).

## 4. Discussion

Recent meta-analysis studies have reported that the RT protocol can moderately increase muscle mass in post-menopausal and elderly women but does not reduce fat mass [33]. Flandez et al. reported that among older women, power resistance training with elastic bands for 20 weeks led to significant negative changes in fat mass, fat-free mass, and body fat percentage in the control group [34]. These findings are consistent with our results. Previous studies have recommended weight-bearing or resistance exercise for at least 6 months to improve bone health in older adults [35]. Our study showed that post-test whole-body BMD and forearm BMD were significantly lower than the pre-test values in the CON group, while four-site BMD did not change significantly in the RT group. Bocalini et al. reported that among older women, BMD did not change significantly in participants who underwent resistance training for 24 weeks, but it decreased significantly in the control group [36]. These results are consistent with our results. Nevertheless, the effect on BMD has been reported to differ slightly according to the type and duration of exercise. Chien et al. found that among osteopenic post-menopausal women, a 24-week aerobic exercise program significantly increased lumbar BMD (2%) and femoral neck BMD (6.8%) in the exercise group, while BMD was decreased in the control group [37]. Thus, a 24-week exercise program could increase or maintain BMD and prevent osteoporosis in older women regardless of the type of exercise.

Resistance exercise training has a positive effect on the functional fitness of older adults [38,39]. In the present study, functional fitness was significantly improved or maintained after 6 months of resistance exercise training in the RT group. In contrast, post-test functional fitness was significantly decreased compared to the pre-test values in the CON group. Oesen et al. reported that among older adults, elastic band resistance training for 24 weeks (twice per week) resulted in a significant increase in lower body strength [38]. Hanson et al. found that older adults who underwent strength training for 22 weeks exhibited significant improvements in lower body strength, walking speed, and agility/dynamic balance [39]. These results suggest that resistance exercise training positively affects functional fitness in older adults. Therefore, resistance training is expected to prevent the decrease in physical performance caused by a reduction in muscle strength and induce a positive effect on independence by improving the daily living ability of older women.

Resistance exercise increases muscle strength, muscle power, and cross-sectional muscle area. Marcell et al. reported a 3–4% annual decrease in knee flexor muscle strength in women aged 48–64 years [40]. Generally, the decrease in muscle strength in adults is more significant than the decrease in muscle mass and muscle quality [41,42]. Therefore, in the management of muscle quality among elderly individuals, increasing only the muscle mass does not necessarily prevent muscle loss [41]. In addition, an imbalance in the muscle strength ratio between the quadriceps femoris and biceps femoris muscles increases the injury rate in the lower extremities [42]. Moreover, an imbalance in muscle strength is a predictor of falls [43]. In the present study, fat-free mass and isokinetic muscle strength were maintained in the RT group but decreased in the control group. Previous studies have reported that resistance training improves isokinetic muscle strength in older women [44,45,46]. Beneke et al. reported that resistance training (90% of 1RM) at 60°/s for 16 weeks increased isokinetic muscle strength (15.2%) in older adults [45]. Rabelo et al. found that progressive resistance training for 24 weeks significantly increased knee extensor peak torque (15.6%) in older women [46]. A recent meta-analysis reported that circuit RT had a moderate and large positive effect on trunk, arm, and lower limb strength [47]. In addition, the increases in strength observed in circuit RT were remarkably more significant than the change observed in the control group [47]. Furthermore, circuit RT improved cardiorespiratory fitness and strength and optimized body composition in middle-aged women and older women [47]. Thus, resistance exercise training can improve or maintain the muscle strength of older women.

## 5. Limitations and Strengths

This study has some limitations. Changes in body composition due to general aging cause a decrease in bone mineral density and muscle mass and an increase in fat mass [48,49]. A decrease in muscle mass and strength increases the risk of fractures, the quality of life decreases, and independent life becomes difficult [50]. In previous studies, musculoskeletal changes according to age negatively affect 7% of the older adults over the age of 70, and the worsening rate increases as the age increases, negatively affecting more than 20% of the older adults until the age of 80 [51]. In addition, muscle strength decreases by 1.5% every year, which accelerates to 3% every year after the age of 60 [52]. The participants in our study were 73 to 89-year-old adults (80.55 ± 4.94 years) with adverse changes in body composition and muscle strength during the intervention period. For this reason, negative changes appeared in all variables in the CON group for 24 weeks. Nevertheless, maintaining fat-free mass and isokinetic muscle strength without negative changes in body composition and muscle strength in the RT group is an outstanding achievement of this study as an effect on exercise intervention.

## 6. Conclusions

We observed that resistance exercise training maintained the fat-free mass, BMD, functional fitness, and isokinetic muscle strength of obese older women. Future studies need to investigate the types, methods, and intensity of various training programs and analyze the biochemical indicators of muscle, fat, and bone-related hormones to determine their relevance.

## Figures and Tables

**Table 1:** Physical characteristics of participants.

Variables	RT	CON	*p* Value
Age (years)	81.6 ± 4.78	79.6 ± 5.19	0.266
Height (cm)	151.33 ± 3.74	153.28 ± 4.74	0.218
Weight (kg)	57.66 ± 8.98	58.26 ± 5.94	0.578
Body fat percentage (%)	35.59 ± 4.22	36.88 ± 2.50	0.308

Note. Values are expressed as means ± standard deviations. RT = resistance exercise training, CON = control.

**Table 2:** Twenty-four-week resistance exercise training program for the study.

Program	Contents		Intensity	Frequency
Warm-up	Dynamic and static stretching		-	2 sessions/week
Main exercise	Upper body	Lower body	OMNI Resistance for active muscle scale: 7–9 range Resting time per set: 90 s Yellow band (3 set) 10 Rep (1–4 weeks) 12 Rep (5–8 weeks) 15 Rep (9–12 weeks) Red band (3 set) 10 Rep (13–16 weeks) 12 Rep (17–20 weeks) 15 Rep (21–24 weeks)	
	Abdominal curl-up Biceps curl Chest press Front shoulder raise Lateral shoulder raise Seated row Triceps extension	Calf raises Chair squat Hip extension Hip flexion Hip abduction Hip adduction Toe raise		
Cool down	Static stretching		-	

**Table 3:** Changes of body composition and BMD between pre- and post-tests in obese older women.

Variables	RT			CON			F-Value (η_p_^2^)		
	Pre (95% CI)	Post (95% CI)	Mean Change (95% CI)	Pre (95% CI)	Post (95% CI)	Mean Change (95% CI)	Time	Group	Interaction
Fat mass (kg)	20.74 ± 5.47 (17.71–23.77)	20.13 ± 5.28 (17.20–23.06)	−0.61 (−1.46–0.24)	21.45 ± 2.84 (19.94–22.97)	22.61 ± 2.84 (21.09–24.12)	1.15 *** (0.77–1.53)	1.620 (0.053)	1.108 (0.037)	17.205 (0.372) ^†††^
Fat-free mass (kg)	35.15 ± 4.17 (32.85–37.46)	35.22 ± 3.97 (33.02–37.42)	0.07 (−0.43–0.57)	35.03 ± 3.53 (33.15–36.90)	34.45 ± 3.31 (32.68–36.21)	−0.58 *** (−0.89–−0.27)	3.586 (0.110)	0.114 (0.004)	5.700 (0.164) ^†^
Body fat percentage (%)	35.59 ± 4.22 (33.26–37.93)	34.83 ± 4.03 (32.60–37.07)	−0.76 (−1.64–0.12)	36.88 ± 2.50 (35.54–38.21)	38.46 ± 2.42 ^#^ (37.17–39.75)	1.59 *** (1.19–1.98)	3.499 (0.108)	4.248 (0.128) ^†^	28.266 (0.494) ^†††^
Whole-body BMD (g/cm^2^)	1.00 ± 0.21 (0.89–1.12)	1.01 ± 0.20 (0.90–1.13)	0.01 (0–0.02)	0.97 ± 0.14 (0.89–1.04)	0.96 ± 0.14 (0.88–1.03)	−0.01 *** (−0.02–−0.01)	0.020 (0.001)	0.567 (0.019)	12.385 (0.299) ^†††^
Femur BMD (g/cm^2^)	0.73 ± 0.11 (0.67–0.79)	0.72 ± 0.12 (0.66–0.79)	−0.01 (−0.03–0.01)	0.69 ± 0.10 (0.63–0.74)	0.68 ± 0.10 (0.62–0.73)	−0.01 (−0.03–0.01)	3.079 (0.096)	1.519 (0.050)	0.074 (0.003)
Lumbar BMD (g/cm^2^)	0.81 ± 0.14 (0.74–0.89)	0.82 ± 0.15 (0.74–0.90)	0.01 (−0.03–0.04)	0.80 ± 0.12 (0.74–0.87)	0.81 ± 0.11 (0.76–0.87)	0.01 (−0.01–0.03)	0.534 (0.018)	0.015 (0.001)	0.027 (0.001)
Forearm BMD (g/cm^2^)	0.42 ± 0.05 (0.39–0.45)	0.42 ± 0.05 (0.39–0.45)	0 (0–0.01)	0.42 ± 0.05 (0.39–0.45)	0.41 ± 0.05 (0.38–0.44)	−0.01 ** (−0.01–0)	5.065 (0.149) ^†^	0.288 (0.010)	6.228 (0.177) ^†^

Note. Values are expressed as means ± standard deviations. CI = confidence interval, RT = resistance exercise training, CON = control, BMD = bone mineral density. Significant interaction or main effect: ^†^ *p* < 0.05, ^†††^ *p* < 0.001; significant difference between pre- and post-test: ** *p* < 0.01, *** *p* < 0.001; significant difference between RT and CON groups: ^#^ *p* < 0.05.

**Table 4:** Changes of functional fitness between pre- and post-tests in obese older women.

Variables	RT			CON			F-Value (η_p_^2^)		
	Pre (95% CI)	Post (95% CI)	Mean Change (95% CI)	Pre (95% CI)	Post (95% CI)	Mean Change (95% CI)	Time	Group	Interaction
Hand grip strength (kg)	21.20 ± 3.53 (19.24–23.16)	22.90 ± 3.34 (21.05–24.75)	1.70 ** (0.71–2.69)	19.84 ± 4.33 (17.53–22.15)	19.03 ± 3.45 ^##^ (37.17–39.75)	−0.81 (−1.76–0.14)	1.926 (0.062)	4.110 (0.124)	15.433 (0.347) ^†††^
Lower body flexibility (cm)	22.43 ± 8.68 (17.63–27.24)	24.44 ± 7.98 (20.01–28.86)	2.01 (−0.47–4.48)	20.91 ± 6.99 (17.18–24.63)	17.68 ± 6.88 ^#^ (14.01–21.34)	−3.23 ** (−5.44–−1.02)	0.627 (0.021)	2.474 (0.079)	11.479 (0.284) ^††^
Upper body flexibility (cm)	−11.77 ± 14.17 (−19.61-−3.92)	−11.35 ± 15.85 (−20.13-−2.57)	0.42 (−2.46–3.20)	−11.99 ± 12.85 (−18.83-−5.14)	−12.47 ± 10.95 (−18.30–−6.63)	−0.48 (−2.42–1.46)	0.002 (0.000)	0.020 (0.001)	0.330 (0.011)
Lower body strength (n)	15.07 ± 4.22 (12.73–17.40)	18.93 ± 5.69 (15.78–22.08)	3.87 *** (1.95–5.78)	16.19 ± 3.02 (14.58–17.79)	15.13 ± 3.56 ^#^ (13.23–17.02)	−1.06 (−2.46–0.33)	6.522 (0.184) ^†^	0.908 (0.030)	20.154 (0.410) ^†††^
Upper body strength (n)	18.27 ± 4.06 (16.02–20.52)	19.47 ± 4.24 (17.12–21.81)	1.20 (−0.70–3.10)	19.19 ± 5.06 (16.49–21.89)	17.13 ± 4.75 (14.60–19.65)	−2.06 *** (−3.02–−1.10)	0.783 (0.026)	0.206 (0.007)	11.202 (0.279) ^††^
Agility and dynamic balance (s)	6.48 ± 1.42 (5.70–7.27)	6.03 ± 1.06 (5.45–6.62)	−0.45 (−1.16–0.26)	5.90 ± 1.04 (5.34–6.45)	6.44 ± 1.10 (5.85–7.03)	0.54 ** (0.20–0.88)	0.064 (0.002)	0.057 (0.002)	7.532 (0.206) ^††^
Aerobic endurance (n)	109.87 ± 16.72 (100.61–119.13)	113.80 ± 31.12 (96.57–131.03)	3.93 (−12.33–20.19)	107.81 ± 14.75 (99.95–115.67)	99.44 ± 18.01 (89.84–109.03)	−8.38 (−15.11–−1.64)	0.306 (0.007)	1.647 (0.054)	2.351 (0.075)

Note. Values are expressed as means ± standard deviations. CI = confidence interval-RT = resistance exercise training, CON = control. Significant interaction or main effect: ^†^ *p* < 0.05, ^††^ *p* < 0.01, ^†††^ *p* < 0.001; significant difference between pre- and post-test: ** *p* < 0.01, *** *p* < 0.001; significant difference between RT and CON groups: ^#^ *p* < 0.05, ^##^ *p* < 0.01.

**Table 5:** Changes of isokinetic muscle strength at 60°/s between pre- and post-tests in obese older women.

Variables	RT			CON			F-Value (η_p_^2^)		
	Pre (95% CI)	Post (95% CI)	Mean Change (95% CI)	Pre (95% CI)	Post (95% CI)	Mean Change (95% CI)	Time	Group	Interaction
DL extensor peak torque (Nm)	70.87 ± 23.55 (57.82–83.91)	76.20 ± 21.91 (64.07–88.33)	5.33 (−2.25–12.92)	66.88 ± 15.56 (58.58–75.17)	62.06 ± 16.60 (53.22–70.91)	−4.81 * (−8.93-−0.70)	0.017 (0.001)	1.801 (0.058)	6.562 (0.185) ^†^
DL extensor peak torque %BW (%)	126.27 ± 46.52 (100.51–152.03)	139.73 ± 48.53 (112.86–166.61)	13.47 (−4.01–30.94)	116.81 ± 28.65 (101.54–132.08)	109.50 ± 31.92 ^#^ (92.49–126.51)	−7.31 * (−13.94–−0.68)	0.523 (0.018)	2.137 (0.069)	5.962 (0.171) ^†^
NDL extensor peak torque (Nm)	69.20 ± 21.37 (57.36–81.04)	75.53 ± 25.40 (61.47–89.60)	6.33 (−2.66–15.32)	66.38 ± 14.53 (58.63–74.11)	64.13 ± 13.54 (56.91–71.34)	−2.25 (−6.59–2.09)	0.799 (0.027)	1.197 (0.040)	3.531 (0.109)
NDL extensor peak torque %BW (%)	122.33 ± 40.88 (99.69–144.97)	135.53 ± 48.97 (108.41–161.65)	13.20 * (−1.33–27.73)	113.81 ± 24.60 (100.70–126.92)	110.63 ± 25.78 (96.89–124.36)	−3.19 (−10.94–4.57)	1.757 (0.057)	1.802 (0.059)	4.706 (0.140) ^†^
DL flexor peak torque (Nm)	31.13 ± 12.82 (24.04–38.23)	35.00 ± 9.70 (29.63–40.37)	3.87 * (0.50–7.23)	29.56 ± 15.02 (21.56–37.57)	28.06 ± 12.73 (21.28–34.84)	−1.50 (−4.16–1.16)	1.410 (0.046)	0.904 (0.030)	7.251 (0.200) ^†^
DL flexor peak torque %BW (%)	54.80 ± 22.56 (42.31–67.29)	62.40 ± 18.48 (52.17–72.63)	7.60 * (1.05–14.15)	50.88 ± 27.01 (36.48–65.27)	46.94 ± 22.51 ^#^ (34.94–58.93)	−3.94 (−9.47–1.59)	0.843 (0.028)	1.472 (0.048)	8.361 (0.224) ^††^
NDL flexor peak torque (Nm)	32.33 ± 15.92 (23.52–41.15)	35.13 ± 13.60 (27.60–42.67)	2.80 (−0.26–5.86)	28.56 ± 16.65 (19.69–37.43)	26.19 ± 12.10 ^#^ (19.74–32.64)	−2.38 (−5.88–1.13)	0.038 (0.001)	1.519 (0.050)	5.598 (0.162) ^†^
NDL flexor peak torque %BW (%)	57.53 ± 29.06 (41.44–73.62)	62.93 ± 26.01 (48.53–77.34)	5.40 (−0.62–11.42)	49.94 ± 30.52 (33.67–66.20)	44.50 ± 23.71 ^#^ (31.86–57.14)	−5.44 (−11.43–0.55)	0.000 (0.000)	1.814 (0.059)	7.429 (0.204) ^†^

Note. Values are expressed as means ± standard deviations. CI = confidence interval, RT = resistance exercise training, CON = control, DL = dominant leg, NDL = non-dominant leg, BW = body weight. Significant interaction or main effect: ^†^ *p* < 0.05, ^††^ *p* < 0.01; significant difference between pre- and post-test: * *p* < 0.05; significant difference between RT and CON groups: ^#^ *p* < 0.05.

**Table 6:** Changes of isokinetic muscle strength at 180°/s between pre- and post-tests in obese older women.

Variables	RT			CON			F-Value (η_p_^2^)		
	Pre (95% CI)	Post (95% CI)	Mean Change (95% CI)	Pre (95% CI)	Post (95% CI)	Mean Change (95% CI)	Time	Group	Interaction
DL extensor peak torque (Nm)	40.67 ± 11.88 (34.09–47.24)	44.13 ± 13.34 (36.75–51.52)	3.47 (−0.74–7.67)	39.75 ± 10.51 (34.15–45.35)	37.56 ± 10.28 (32.09–43.04)	−2.19 * (−4.97–0.59)	0.302 (0.010)	0.886 (0.030)	5.902 (0.169) ^†^
DL extensor peak torque %BW (%)	72.47 ± 23.62 (59.39–85.54)	78.53 ± 26.06 (64.10–92.97)	6.07 (−1.09–13.23)	68.69 ± 17.55 (59.34–78.04)	63.63 ± 17.74 (54.17–73.08)	−5.06 * (−10.44–0.31)	0.059 (0.002)	1.586 (0.052)	7.193 (0.199) ^†^
NDL extensor peak torque (Nm)	39.67 ± 11.46 (33.32–46.01)	41.87 ± 15.09 (33.51–50.23)	2.20 (−3.23–7.63)	36.88 ± 8.52 (32.34–41.41)	35.00 ± 8.66 (30.38–39.62)	−1.88 (−3.71–−0.04)	0.016 (0.001)	1.616 (0.053)	2.449 (0.078)
NDL extensor peak torque %BW (%)	70.20 ± 22.55 (57.71–82.69)	75.87 ± 29.41 (59.58–92.15)	5.67 (−3.52–14.85)	63.56 ± 14.58 (55.79–71.33)	58.50 ± 13.78 (51.15–65.85)	−5.06 * (−8.83–−1.29)	0.018 (0.001)	2.816 (0.089)	5.618 (0.162) ^†^
DL flexor peak torque (Nm)	19.93 ± 7.81 (15.61–24.26)	20.60 ± 7.06 (16.69–24.51)	0.67 (−2.33–3.66)	18.75 ± 7.46 (14.77–22.73)	16.56 ± 6.96 (12.86–20.27)	−2.19 (−3.86–−0.51)	0.931 (0.031)	1.080 (0.036)	3.279 (0.102)
DL flexor peak torque %BW (%)	35.60 ± 15.38 (27.09–44.11)	36.40 ± 13.07 (29.16–43.64)	0.80 (−5.27–6.87)	33.19 ± 13.66 (25.91–40.47)	27.38 ± 13.14 (20.37–34.38)	−5.81 ** (−9.34–−2.29)	2.412 (0.077)	1.480 (0.049)	4.197 (0.126) ^†^
NDL flexor peak torque (Nm)	22.33 ± 10.39 (16.58–28.09)	23.60 ± 9.72 (18.22–28.98)	1.27 (−0.36–2.89)	20.81 ± 9.56 (15.72–25.91)	18.63 ± 7.21 (14.78–22.47)	−2.19 * (−4.16–−0.21)	0.582 (0.020)	0.983 (0.033)	8.183 (0.220) ^††^
NDL flexor peak torque %BW (%)	39.93 ± 20.43 (28.62–51.24)	42.33 ± 19.01 (31.81–52.86)	2.40 (−0.28–5.08)	36.56 ± 17.33 (27.33–45.80)	30.75 ± 12.51 (24.08–37.42)	−5.81 ** (−9.79–−1.83)	2.246 (0.072)	1.462 (0.048)	13.009 (0.310) ^†††^

Note. Values are expressed as means ± standard deviations. CI = confidence interval, RT = resistance exercise training, CON = control, DL = dominant leg, NDL = non-dominant leg, BW = body weight. Significant interaction or main effect: ^†^ *p* < 0.05, ^††^ *p* < 0.01, ^†††^ *p* < 0.001; significant difference between pre- and post-test: * *p* < 0.05, ** *p* < 0.01.
